# Use of Cadaveric Pericardial Tissue in the Surgical Treatment of Neurogenic Bladder

**DOI:** 10.1155/2019/6182397

**Published:** 2019-07-15

**Authors:** Madison Caja, Michaela Lamonde, John Barnard, Stanley Zaslau, Robert E. Shapiro

**Affiliations:** ^1^West Virginia University School of Medicine, Morgantown, WV 26506, USA; ^2^Department of Obstetrics & Gynecology, West Virginia University School of Medicine, Morgantown, WV 26506, USA; ^3^Department of Urology, West Virginia University School of Medicine, Morgantown, WV 26506, USA

## Abstract

The surgical treatments for neurogenic bladder are extremely variable. The lack of specific treatment guidelines makes this disease process even more challenging to treat. We present a case of a 55-year-old female with neurogenic bladder secondary to spinal cord injury (SCI). Her incontinence was conservatively managed with indwelling Foley drainage. Despite continued upsizing of the Foley catheters, the patient continued to have urinary leakage. The patient subsequently underwent a transvaginal bladder neck closure (BNC) with suprapubic bladder neck diversion (SPC). The urethra was successfully closed and uniquely supported with the use of cadaveric pericardial tissue (CPT). This surgical approach of neurogenic bladder provides durable continence with short operative times, minimal patient morbidity, decreased hospital length, and low risk of progressive renal dysfunction. BNC with SPC can provide an excellent management solution for neurogenic bladder from spinal cord injury refractory to conservative management.

## 1. Introduction

Spinal cord injuries (SCI) arising from trauma or surgical complications are an unfortunate common occurrence with approximately 54 per million per year in the United States [1]. The sequalae that occur after SCI can often be severe. One of the most common complications from SCI is voiding dysfunction [2]. SCI causes loss of both motor and sphincter control of the bladder. As a result, patients often require intermittent catheterization or placement of indwelling catheters. These treatments, however, are not always curative. Patients with even the most intensive bladder regimens will often suffer from urinary incontinence.

For patients with intractable incontinence despite indwelling catheters, bladder neck closure (BNC) with suprapubic catheter placement (SPC) is a treatment option. Limited published data exist on this treatment option and the data that exists primarily involves an abdominal surgical approach. Moreover, this data also suggests that complications such as bladder outlet fistulae can occur in 20–30 % of patients [3].

We present a case of a patient with neurogenic bladder that underwent a transvaginal BNC with SPC diversion. The urethra was successfully closed and uniquely supported with the use of cadaveric pericardial tissue (CPT) to prevent fistula formation.

## 2. Case Report

A 55-year-old female suffered a spinal cord injury (SCI) following a spinal surgery in 2015 and consequently became wheel chair bound. Her health history was significant for morbid obesity, type 2 diabetes, hypertension, chronic obstructive pulmonary disease (COPD), history of deep venous thrombosis (DVT), recurrent urinary tract infections (UTIs), and sacral decubitus ulcers. The patient was treated by the urology department for kidney stones prior to her SCI. Her past surgical history was significant for pelvic laparoscopy, tubal ligation, exploratory laparotomy, and colostomy placement.

The patient's urinary incontinence was initially managed conservatively with Foley catheters and anticholinergic medications. She required multiple upsizing of catheters due to continued urinary leakage. The persistent urinary incontinence was significantly affecting the patient's quality of life and was further complicating her sacral decubitus ulcers. The patient elected to proceed with BNC with SPC diversion. Secondary to medical comorbidities and history of prior abdominal surgery, the decision was made to use a transvaginal approach. This case was a combined surgical approach with urology and urogynecology.

**Figure 1:**
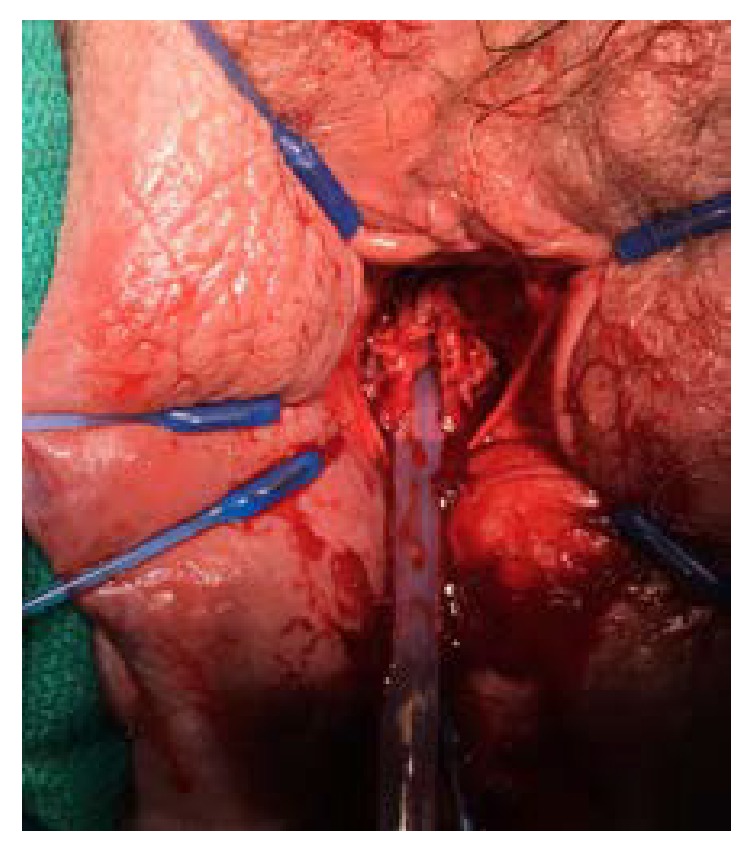
Patulous urethra is clearly observed circumferentially surrounding the Foley catheter.

**Figure 2:**
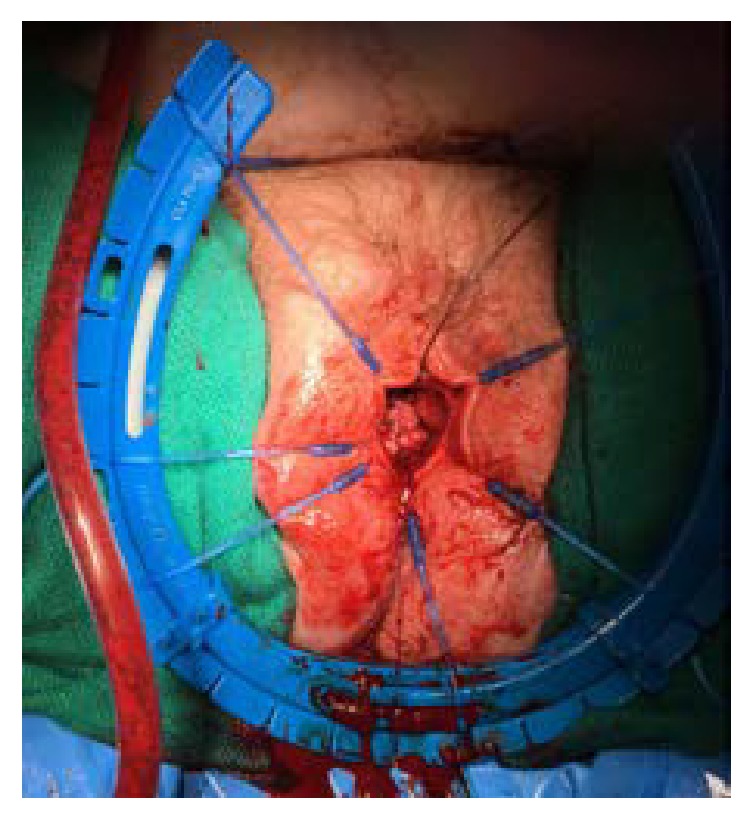
The bladder neck closure is seen with both the interrupted and imbricating layer of 2-0 vicryl closed.

**Figure 3:**
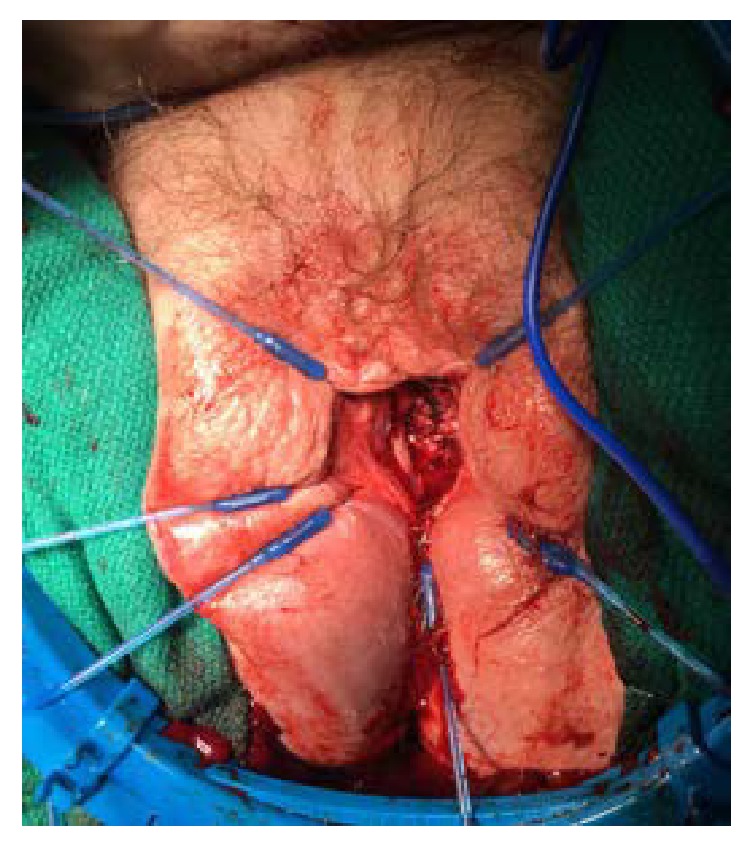
The third layer of the bladder neck closure can be appreciated. The cadaveric pericardial tissue in place to prevent vesicovaginal fistula formation.

**Figure 4:**
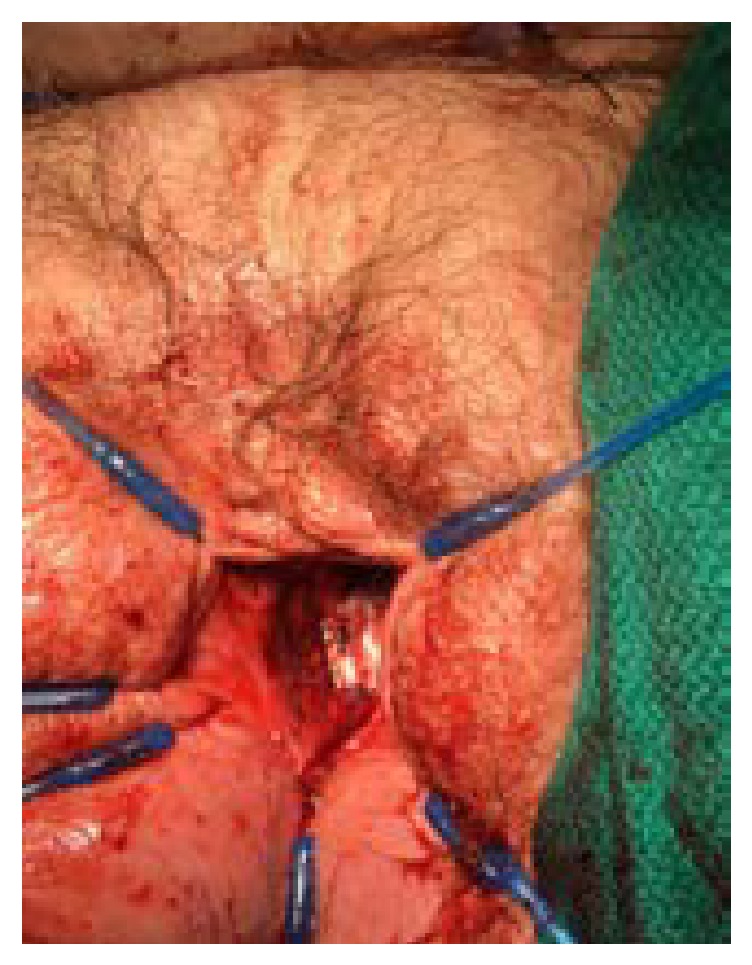
Final layer of bladder neck closure with the cadaveric pericardial tissue in place.

Once in the operating room, the full defect of the urethra was appreciated (Figure 1). The urethra was patulous with significant erosions on the dorsal side. The urethra was dissected and mobilized to the level of the bladder neck. The bladder neck was then closed in four layers. An interrupted layer followed by imbricating layers of 2-0 vicryl was used (Figure 2). A layer of cadaveric pericardial tissue (CPT) was incorporated in the bladder neck closure for enhanced support and to prevent the occurrence of fistula (Figures 3 and 4). Finally, the vaginal mucosa was closed with running 3-0 vicryl. A SPC was then placed. The patient tolerated the procedure well and was discharged to home the same day.

The patient has been seen for SPC exchange at 3 and 6 months postoperatively. She is doing well and has no further incontinence. Her decubitus ulcers have greatly improved.

## 3. Discussion

Since 1985, there have been only a handful of published studies that describe transvaginal bladder neck closure (BNC) and suprapubic catheter (SPC) placement in the treatment of neurogenic bladder [4]. Most of these studies cite bladder outlet fistulae as a significant complication [3]. Our case is unique with the usage of cadaveric pericardial tissue (CPT) to augment the closure and thereby reduce the risk of fistula formation. One study successfully combated the risk of fistula formation by using a posterior urethral flap to secure the suture line [5]. In our case, the decision was made to use CPT to help augment the native tissue repair secondary to the concern over the patient's medical comorbidities interfering with wound healing. Due to the low cost, ease of handling, and less immunogenicity, we feel that CPT is a good option to augment native tissue repairs [6]. A paucity of studies exist on biologic grafts used to prevent fistula formation. However, literature from colon and rectal surgery reports good success with utilizing porcine submucosa to close fistula tracts of the anus [7].

The traditional abdominal approach of a BNC and SPC has a reported 15% complication rate and average length of stay of 4 days [3]. Our approach offers an excellent alternative for patients who are poor candidates for abdominal surgery. We were able to safely send our patient home on postoperative day 0 and without the risk of a significant abdominal wound infection or further abdominal surgery. It has been shown that transvaginal BNC, compared to abdominal approach, is associated with decreased short-term complications and additionally both a shorter operative time and hospital stay [8].

The chronic use of an indwelling catheter, irrespective of whether it travels via the urethra or via the suprapubic route, has been associated with a number of undesired and cumbersome problems such as irritation, leakage, infections, stone formation, catheter dislocation, cuff rupture, and metaplastic malformation [9]. Another option for these patients could instead be a catheterizable vesicostoma in combination with a bladder neck closure. Unfortunately, the most common reported complication of catheterizable vesicostomas is incontinence. According to the most recent literature, this can be as high as 30 percent [10]. In addition, approximately 20 percent of these patients require reoperation for stomal stenosis [10]. As this patient already suffered from intractable incontinence and had significant medical comorbidities that prohibited major surgery, we elected to proceed with SPC.

Another consideration in this case would have been to perform a Martius modified labial fat pad flap (MMLFPF), commonly known as the Martius flap. The Martius flap has been described in the literature most commonly for vaginal fistula repair [11]. This technique has been noted to be successful in cases where vaginal tissue integrity is a concern [12]. We chose cadaveric pericardial tissue over a MMLFPF to diminish risk of potential long term patient sequelae such as labial pain, numbness, and distortion, all of which have been described in the literature [13].

We hope this case report adds to a body of evidence in support of CPT in tissue closure and negates the risk of fistulas. This case also highlights the importance of a multidisciplinary team and the benefit of combined surgical cases when appropriate. By combining skills of gynecologic and urologic surgeons, we were able to safely and efficiently treat a major problem for our patient.

## 4. Conclusion

Bladder neck closure with suprapubic catheter should be considered in patients with neurogenic bladder refractory to conservative treatment. The transvaginal approach, with the adjunct use of cadaveric pericardial tissue (CPT), offers comparable outcomes to the abdominal approach with considerably less morbidity. The use of CPT for tissue interposition potentially decreases the risk of failure and vesicovaginal fistula formation, although more research is needed on this subject. Using a multidisciplinary surgical approach, both urologists and urogynecologists should be made aware of this option.
